# Impaired neural pathway in gastric muscles of patients with diabetes

**DOI:** 10.1038/s41598-018-24147-y

**Published:** 2018-05-08

**Authors:** Yang Won Min, Eun-Ju Ko, Ji-Yeon Lee, Poong-Lyul Rhee

**Affiliations:** 1 Department of Medicine, Sungkyunkwan University School of Medicine, Seoul, Korea; 2 Biomedical Research Institute, Samsung Medical Center, Sungkyunkwan University School of Medicine, Seoul, Korea

## Abstract

To explore the pathogenic mechanism of diabetic gastropathy, we investigated differences in response to electrical field stimulation (EFS) of gastric muscles from diabetic and non-diabetic (control) patients. Gastric specimens were obtained from 34 patients and 45 controls who underwent gastrectomy for early gastric cancer. Using organ bath techniques, we examined peak and nadir values of contraction under EFS. To examine responses to purinergic and nitrergic inhibition without cholinergic innervation, atropine, MRS2500, and N-nitro-L-arginine (L-NNA) were added sequentially to the organ bath. Tetrodotoxin (TTX) was used to confirm that the responses to EFS were mediated via neural stimulation. In the absence of pharmacological agents, peak contraction amplitude was greater in non-diabetic controls compared to diabetics only in the distal longitudinal gastric muscles. However, the nadir was greater in controls than in patients in both proximal and distal gastric circular muscles. Addition of MRS2500 could not decrease the nadir in both controls and patients, both in the proximal and distal stomach. However, L-NNA completely reversed the relaxation. TTX had no further effect on nadir. In conclusion, impaired inhibitory nitrergic neural pathway in both proximal and distal stomach and impaired excitatory cholinergic neural pathway in the distal stomach may contribute to the pathogenic mechanism underlying diabetic gastropathy.

## Introduction

Diabetes mellitus (DM) is associated with an increased prevalence of upper gastrointestinal symptoms. Symptoms attributable to gastroparesis are reported in 5 to 12% of patients with DM^1,2^. Gastroparesis is characterized by abnormal gastric function resulting in delayed gastric emptying^3^. Pathogenic mechanisms proposed to underlie delayed gastric emptying in DM include autonomic neuropathy, loss of neuronal nitric oxide synthase (nNOS) expression, loss of interstitial cells of Cajal (ICC), low level of antioxidant heme oxygenase-1, and loss of CD206-positive anti-inflammatory macrophages^4–15^.

Loss of nNOS expression and loss of ICC are the most consistent findings in diabetic gastroparesis^16–18^. Loss of nNOS itself may not lead to a development of delayed gastric emptying as sildenafil does not improve gastric emptying in patients with diabetic gastroparesis^19^. In streptozotocin-induced diabetic rats, nitrergic gastric relaxation correlates better with the active dimeric form of nNOS rather than absolute nNOS levels^20^. Thus, post-translational modification may be important. Loss of ICC correlates with development of delayed gastric emptying in humans with diabetic gastroparesis as well as in animal models^6,17,18,21,22^. In a retrospective study using paraffin-embedded gastric specimens, decreased expression of nNOS and substance P (SP) was accompanied by loss of ICC levels in patients with DM^6^. Gastroparesis Clinical Research Consortium study using prospectively collected specimens also reported that loss of ICC is associated with delayed gastric emptying^17^. Immune cells also play a role in the development of diabetic gastroparesis. CD206-positive HO-1 expressing M2 macrophages show a protective effect against diabetic gastroparesis in mice^14^. A recent human study also demonstrates a correlation between ICC and CD206-positive anti-inflammatory macrophage numbers suggesting that loss of ICC may be the result of loss of the macrophage subtype^15^.

The studies mentioned above have focused on cellular abnormalities. However, gastric emptying relies on coordinated actions of the proximal and distal stomach, which requires the interplay of the nervous system, smooth muscle, ICCs, and immune cells. Little has been studied on the changes of gastric smooth muscle contractility according to the region of the stomach. In our previous study, we reported that acetylcholine has varying effects on human gastric contractions according to the stomach region^23^. Based on these observations, we investigated whether DM would affect the contractility of the human stomach with regional differences^24^. Comparison of spontaneous gastric muscle strip contractions between diabetic patients and control subjects has shown significant differences between them. The diabetic gastric muscle has higher basal tone in the distal stomach than in control muscle, lower frequency of contraction in the distal stomach, and less acetylcholine-induced positive inotropic effect in the proximal stomach. These changes of contractility of diabetic stomach may play a role in pathogenesis of diabetic gastroparesis. To further characterize the contractility of diabetic stomach, we investigated differences in response to electrical field stimulation (EFS) of human gastric muscles between diabetic patients and control subjects.

## Results

### EFS-induced responses in circular muscle strips

**Figure 1:**
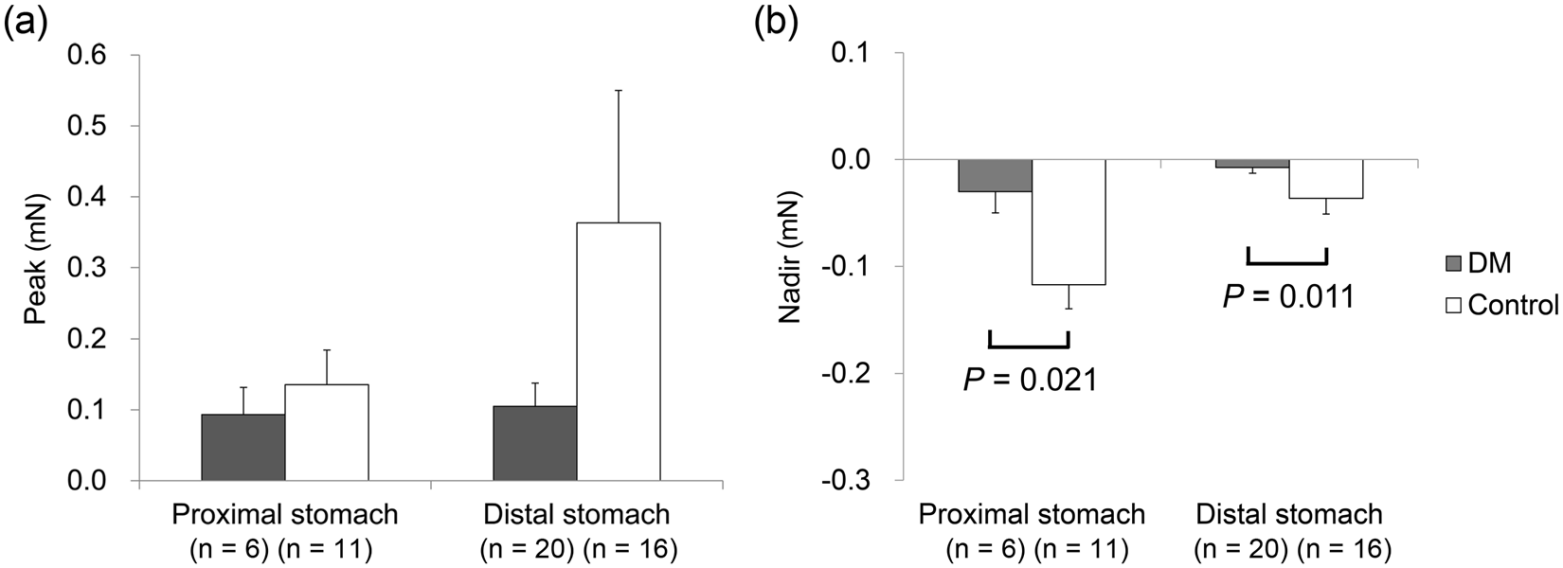
Electrical field stimulation (EFS)-induced response of gastric circular muscle strips in the control state: (**a**) The peak did not differ between diabetic patients and control subjects. (**b**) The nadir was greater in control subjects than in diabetic patients.

Figure 1 shows EFS-induced response of the gastric circular muscle strips in the control state. The peak did not differ between diabetic patients and control subjects in both the proximal and distal stomach. However, the nadir was greater in control subjects than in diabetic patients in both the proximal and distal stomach (−0.12 ± 0.02 mN vs. −0.03 ± 0.02 mN, p = 0.021 and −0.04 ± 0.01 mN vs. −0.01 ± 0.01 mN, p = 0.011, respectively).

**Figure 2:**
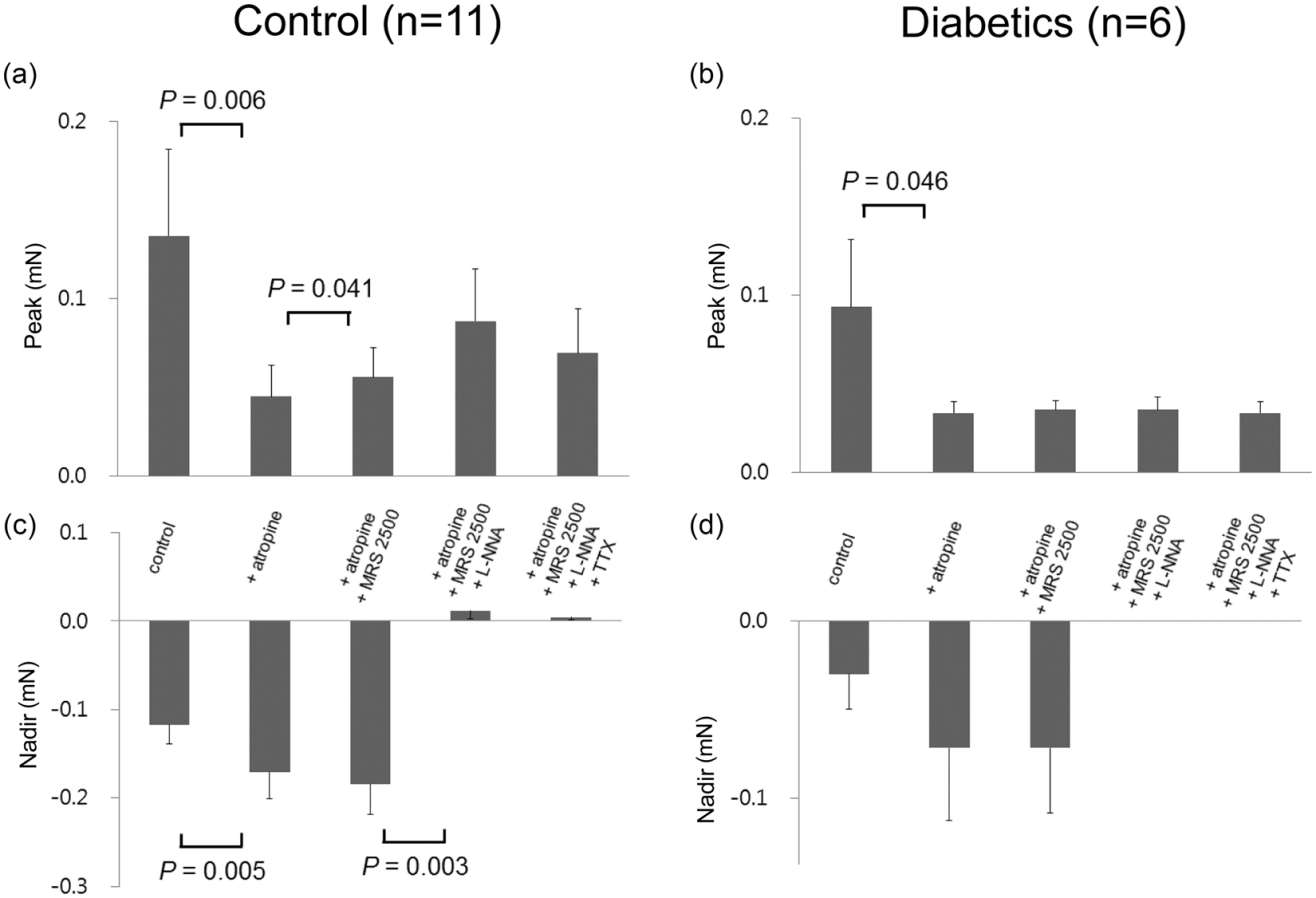
Electrical field stimulation (EFS)-induced response of proximal gastric circular muscle strips after serial administration of atropine, MRS2500, N-nitro-L-arginine (L-NNA), and tetrodotoxin (TTX). (**a**) Atropine decreased the peak and MRS2500 increased the peak in the control subjects. (**b**) Atropine decreased the peak in diabetic patients. (**c**) Atropine increased the nadir and L-NNA abolished relaxation in the control subjects. (**d**) Also in the diabetic patients, L-NNA decreased the nadir with marginal statistical significance (p = 0.080). The Wilcoxon signed-rank test was used to evaluate the effects of each drug by compare values to the previous one.

Figure 2 shows EFS-induced response of the proximal gastric circular muscle strips after serial administration of atropine (a muscarinic antagonist, 1 µM), N-nitro-L-arginine (L-NNA, a NO synthase inhibitor, 100 µM), MRS2500 (a purinergic P_2_Y_1_ antagonist, 1 µM), and tetrodotoxin (TTX, 1 µM). When atropine was added, the peak was decreased due to inhibition of contraction in both the control subjects and diabetic patients from 0.14 ± 0.05 mN to 0.04 ± 0.02 mN (p = 0.006) and 0.09 ± 0.04 mN to 0.03 ± 0.01 mN (p = 0.046), respectively. Except for the addition of MRS2500, which increased the peak in the control subjects from 0.05 ± 0.02 mN to 0.06 ± 0.02 mN (p = 0.041), addition of MRS2500, L-NNA, and TTX had no significant effect on the peak in both the control subjects and diabetic patients. Atropine also increased the nadir by inhibiting contraction in the control subjects from −0.12 ± 0.02 mN to −0.17 ± 0.30 mN (p = 0.005). Addition of MRS2500 had no effect on the nadir in both the control subjects and diabetic patients. However, L-NNA decreased the nadir by abolishing relaxation in the control subjects from −0.18 ± 0.03 mN to 0.01 ± 0.01 mN (p = 0.003). Also, in the diabetic patients, L-NNA decreased the nadir from −0.07 ± 0.04 mN to 0.00 ± 0.00 mN with marginal statistical significance (p = 0.080). TTX had no further effect on the nadir in both the control subjects and diabetic patients.

**Figure 3:**
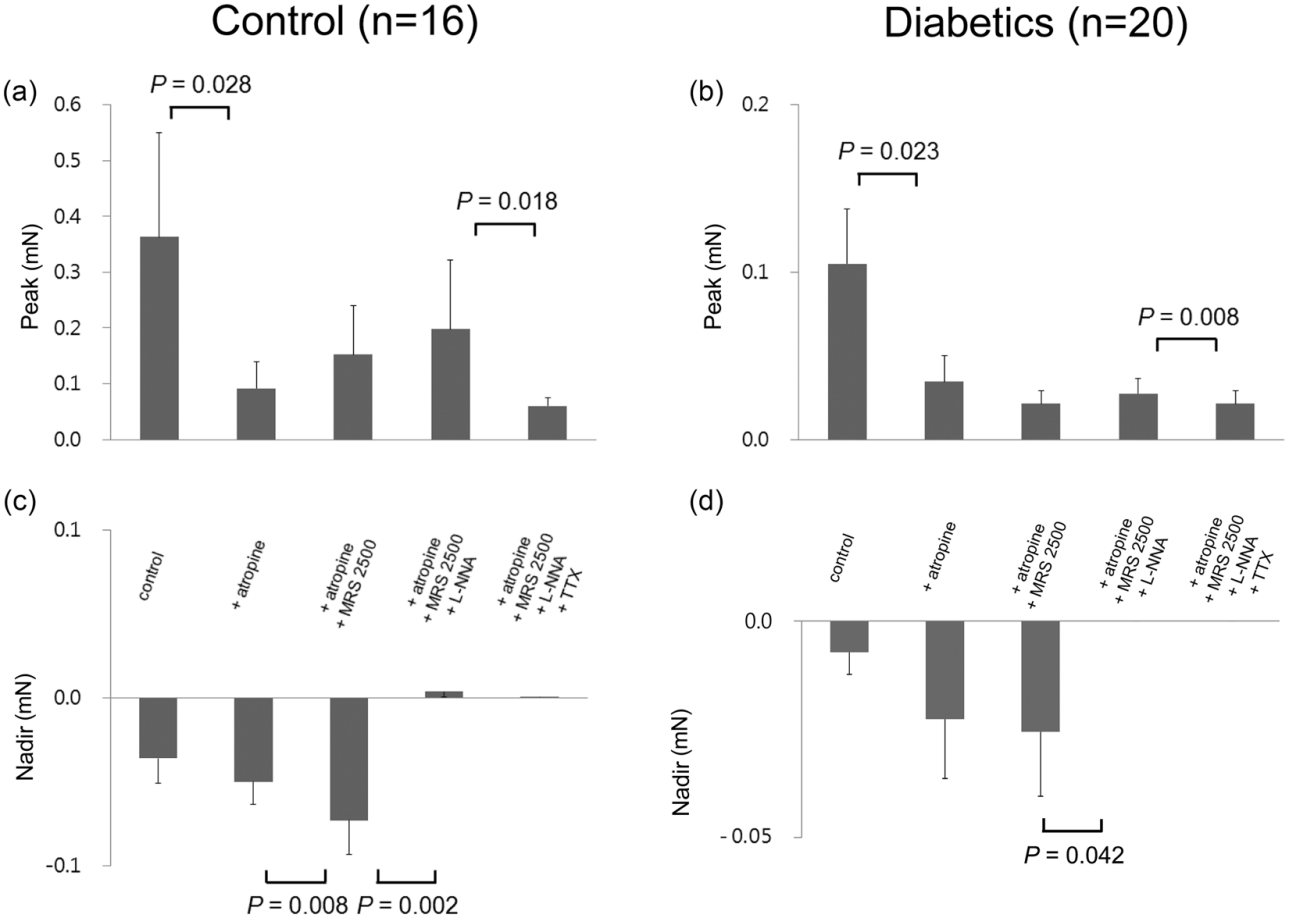
Electrical field stimulation (EFS)-induced response of distal gastric circular muscle strips after serial administration of atropine, MRS2500, N-nitro-L-arginine (L-NNA), and tetrodotoxin (TTX). (**a** and **b**) Atropine decreased the peak and TTX decreased the peak further in both the control subjects and diabetic patients. (**c**) MRS2500 increased the nadir but L-NNA abolished relaxation in the control subjects. (**d**) Also in the diabetic patients, L-NNA abolished relaxation. The Wilcoxon signed-rank test was used to evaluate the effects of each drug by compare values to the previous one.

Figure 3 shows EFS-induced response of the distal gastric circular muscle strips after serial administration of atropine, L-NNA, MRS2500, and TTX. When atropine was added, the peak was decreased due to inhibition of contraction in both the control subjects and diabetic patients from 0.36 ± 0.19 mN to 0.09 ± 0.05 mN (p = 0.028) and 0.11 ± 0.03 mN to 0.03 ± 0.02 mN (p = 0.023), respectively. Addition of MRS2500 and L-NNA had no significant effect on the peak in both the control subjects and diabetic patients. However, addition of TTX decreased the peak in the control subjects and diabetic patients from 0.20 ± 0.12 mN to 0.06 ± 0.02 mN (p = 0.018) and 0.03 ± 0.01 mN to 0.02 ± 0.01 mN (p = 0.008), respectively. Addition of MRS2500 increased the nadir from −0.05 ± 0.01 mN to −0.07 ± 0.02 mN (p = 0.008) in the control subjects but did not have the same effect in diabetic patients. However, L-NNA decreased the nadir by abolishing relaxation in both the control subjects and diabetic patients from −0.07 ± 0.02 mN to 0.00 ± 0.00 mN (p = 0.002) and −0.03 ± 0.02 mN to 0.00 ± 0.00 mN (p = 0.042), respectively. TTX had no further effect on the nadir in both the control subjects and diabetic patients.

### EFS-induced responses in the longitudinal muscle strips

**Figure 4:**
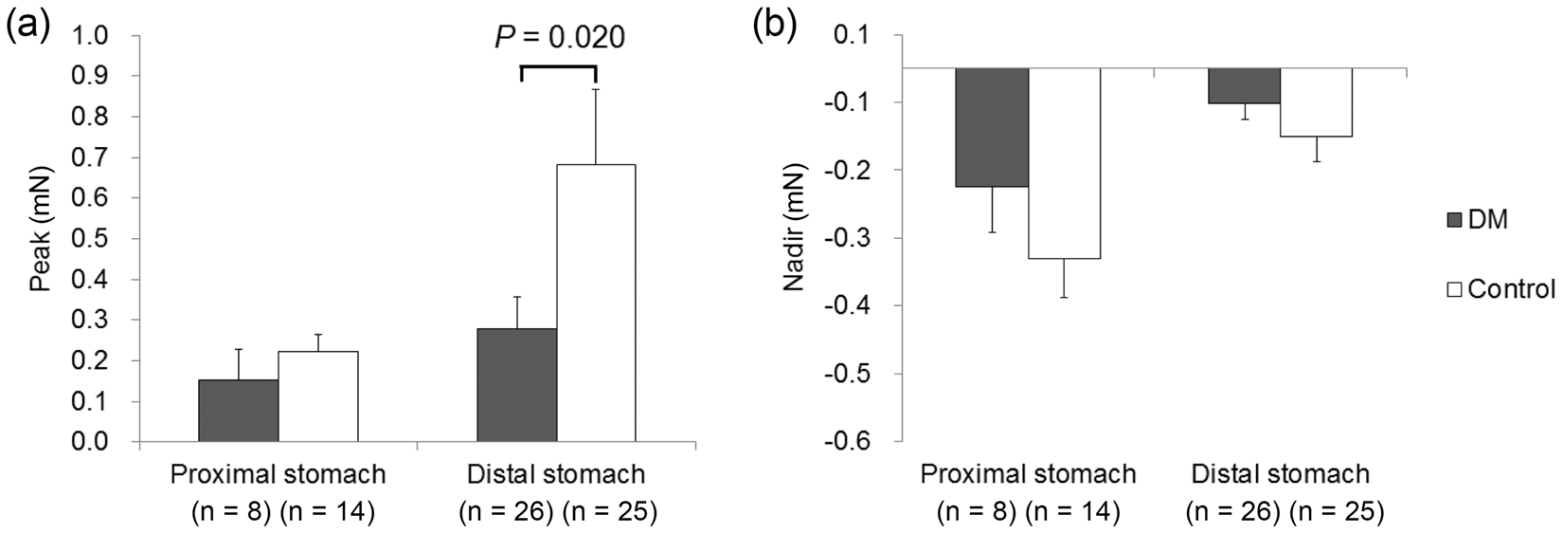
Electrical field stimulation (EFS)-induced response of gastric longitudinal muscle strips in the control state. (**a**) The peak did not differ between diabetic patients and control subjects in the proximal stomach but was greater in control subjects than in diabetic patients in the distal stomach. (**b**) The nadir did not differ between diabetic patients and control subjects in both the proximal and distal stomach.

Figure 4 shows EFS-induced response of the gastric longitudinal muscle strips in the control state. The peak did not differ between diabetic patients and control subjects in the proximal stomach but was greater in control subjects than in diabetic patients in the distal stomach (0.68 ± 0.18 vs. 0.28 ± 0.08, p = 0.020). However, the nadir did not differ between diabetic patients and control subjects in both the proximal and distal stomach.

**Figure 5:**
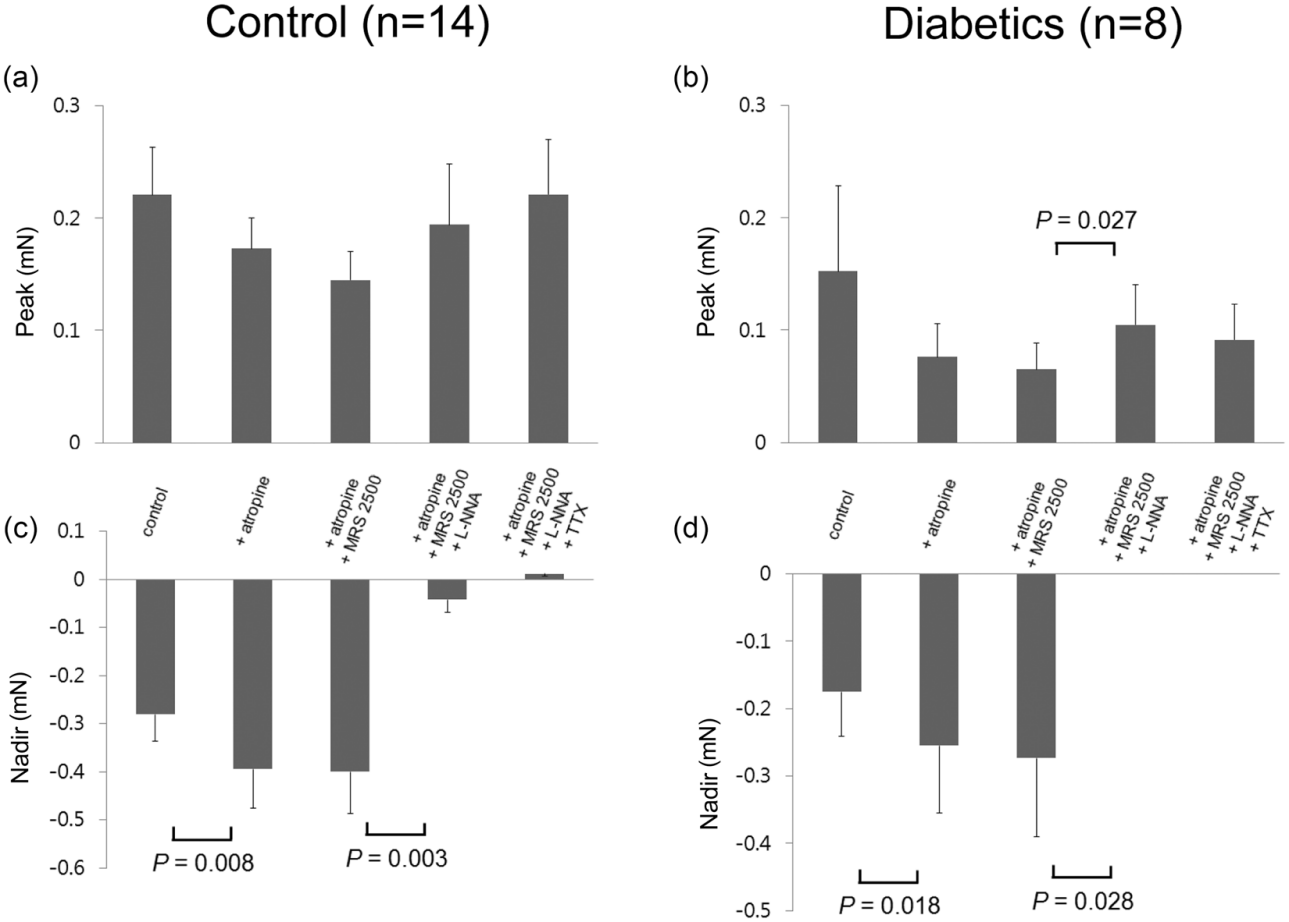
Electrical field stimulation (EFS)-induced response of the proximal gastric longitudinal muscle strips after serial administration of atropine, MRS2500, N-nitro-L-arginine (L-NNA), and tetrodotoxin (TTX). (**a**) No significant effects of serial administration of drugs in the control subjects. (**b**) L-NNA increased the peak in the diabetic patients. (**c**) Atropine increased the nadir but L-NNA abolished relaxation. (**d**) Also in the diabetic patients, atropine increased the nadir but L-NNA abolished relaxation. The Wilcoxon signed-rank test was used to evaluate the effects of each drug by compare values to the previous one.

Figure 5 shows EFS-induced response of the proximal gastric longitudinal muscle strips after serial administration of atropine, L-NNA, MRS2500, and TTX. Except for the addition of L-NNA, which increased the peak in the diabetic patients from 0.07 ± 0.02 mN to 0.11 ± 0.04 mN (p = 0.027), addition of atropine, MRS2500, L-NNA, and TTX had no significant effect on the peak in both the control subjects and diabetic patients. Atropine increased the nadir by inhibiting contraction in both the control subjects and diabetic patients from −0.28 ± 0.06 mN to −0.40 ± 0.08 mN (p = 0.008) and −0.18 ± 0.07 mN to −0.26 ± 0.10 mN (p = 0.018), respectively. Addition of MRS2500 had no effect on the nadir in both the control subjects and diabetic patients. However, L-NNA decreased the nadir by abolishing relaxation in both the control subjects and diabetic patients from −0.40 ± 0.09 mN to −0.04 ± 0.03 mN (p = 0.003) and −0.27 ± 0.12 mN to 0.00 ± 0.00 mN (p = 0.028), respectively. TTX had no further effect on the peak in both the control subjects and diabetic patients.

**Figure 6:**
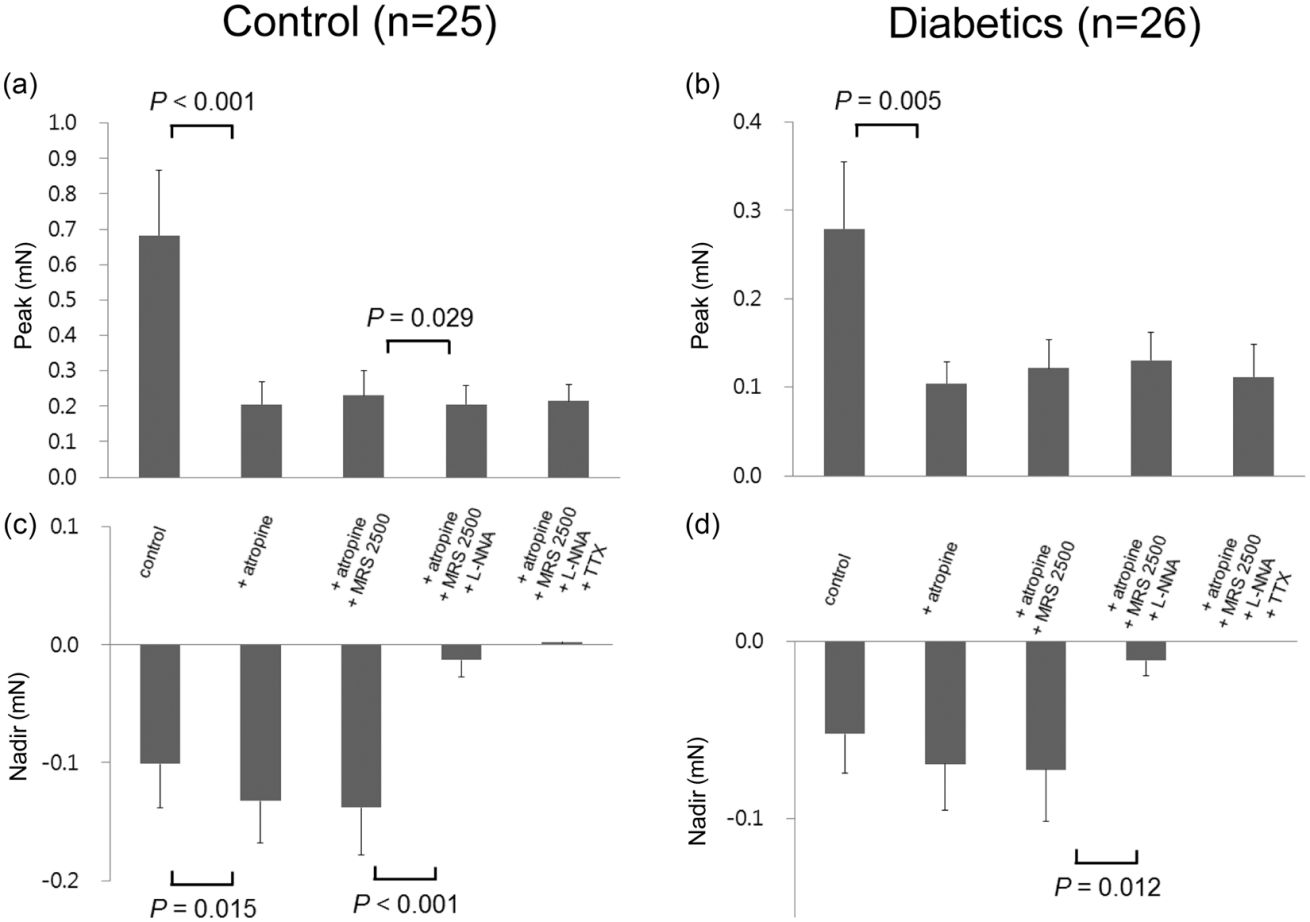
Electrical field stimulation (EFS)-induced response of distal gastric longitudinal muscle strips after serial administration of atropine, MRS2500, N-nitro-L-arginine (L-NNA), and tetrodotoxin (TTX). (**a**) Atropine and L-NNA decreased the peak in the control subjects. (**b**) Atropine decreased the peak in the diabetic patients. (**c**) Atropine increased the nadir but L-NNA abolished relaxation in the control subjects. (**d**) Also in diabetic patients, L-NNA abolished relaxation. The Wilcoxon signed-rank test was used to evaluate the effects of each drug by compare values to the previous one.

Figure 6 shows EFS-induced response of the distal gastric longitudinal muscle strips after serial administration of atropine, L-NNA, MRS2500, and TTX. When atropine was added, the peak was decreased due to inhibition of contraction in both the control subjects and diabetic patients from 0.68 ± 0.18 mN to 0.21 ± 0.06 mN (p < 0.001) and 0.28 ± 0.08 mN to 0.10 ± 0.02 mN (p = 0.005), respectively. Except for the addition of L-NNA, which decreased the peak in the control subjects from 0.23 ± 0.07 mN to 0.21 ± 0.05 mN (p = 0.029), addition of MRS2500, L-NNA, and TTX had no significant effect on the peak in both the control subjects and diabetic patients. Atropine also increased the nadir by inhibiting contraction in the control subjects from −0.10 ± 0.04 mN to −0.13 ± 0.04 mN (p = 0.015). Addition of MRS2500 had no effect on the nadir in both the control subjects and diabetic patients. However, L-NNA decreased the nadir by abolishing relaxation in both the control subjects and diabetic patients from −0.14 ± 0.04 mN to −0.01 ± 0.01 mN (p < 0.001) and −0.07 ± 0.03 mN to −0.01 ± 0.01 mN (p = 0.012), respectively. TTX had no further effect on the nadir in both the control subjects and diabetic patients.

## Discussion

Gastric dysmotility associated with DM causes considerable morbidity including gastroparesis as its most severe form^1,2,25,26^. Gastroparesis not only diminishes quality of life but also increases health-care costs^27,28^. Although understanding the pathogenesis of diabetic gastroparesis at a cellular level has been improved in the past decade, little has been studied on the changes of gastric muscle contractility in humans with DM. In our previous human studies, we demonstrated that acetylcholine has different effects on gastric muscle contractility by the stomach region and DM affects the spontaneous gastric muscle contractility with regional differences^23,24^. To further characterize the change of gastric muscle contractility in a diabetic condition, we investigated differences in response to EFS of human gastric muscles between patients with DM and control subjects.

In our experiments, EFS of the human gastric muscle strips elicited muscle relaxation. In the control state, the nadir was greater in control subjects than in diabetic patients in both the proximal and distal gastric circular muscle strips, but not in the longitudinal muscle strips. These observations indicate that the diabetic stomach has impaired relaxation especially in the circular muscle layer. The different degree of response between the gastric circular and longitudinal muscle strips is not thought to be due to the small sample size of the longitudinal sample because more strips were obtained from the longitudinal muscle. In the canine stomach, density of nerve fibers was higher in the circular muscle than in the longitudinal muscle, and inhibitory nitrergic nerves constituted a substantial fraction of the enteric nervous system^29^. NO and nNOS have been consistently observed to be decreased in animal models and in humans with diabetic gastroparesis. Decreased expression of nNOS in the myenteric plexus of gastric of streptozotocin-diabetic rats, and gastric relaxation is hampered mainly by impaired nNOS expression in the gastric myenteric plexus of spontaneously diabetic rats^16,30^. Iwasaki *et al*. reported loss of nNOS-positive neurons in patients with severe DM, along with loss of ICCs^6^. Taken together, impaired nitrergic neural pathway may contribute to the development of diabetic dysmotility.

To investigate responses to the inhibition of purinergic and nitrergic pathways without cholinergic innervation, MRS2500, and L-NNA were added sequentially to the organ bath in the presence of atropine, and EFS was performed. Addition of MRS2500 could not decrease the nadir. However, L-NNA completely reversed the relaxation by inhibiting nitrergic pathway in both the control subjects and diabetic patients. Although the effect was not statistically significant in the proximal circular muscle strips from the diabetic patients, it might be due to the small sample size (n = 6). Addition of TTX confirms that the EFS-induced relaxation was neurally mediated. These observations indicate that the human gastric relaxation is mainly mediated by nitrergic pathway.

Several neurotransmitters have been suggested to be released by inhibitory neurons, including NO^31–34^, vasoactive intestinal polypeptide^35–38^, and adenosine triphosphate^39,40^. From animal studies, conflicting data exist regarding whether NO predominantly mediates the relaxation^41–43^, or whether NO and vasoactive intestinal polypeptide act as co-transmitters^44–47^. However, NO is considered the major contributor in the non-adrenergic, non-cholinergic relaxation of the human stomach^48–51^, which is consistent with our findings in the present study. Pasricha *et al*. reported that decreased HO-2 immunoreactivity in the stomach of patient with poorly controlled diabetes^7^. HO, the enzyme that gives rise to carbon monoxide, known to regulate neurotransmission and smooth muscle membrane potential^52,53^. The authors suggested that combined loss of both inhibitory molecules may cause a profound disturbance of gastric physiology like gastroparesis^7^. In the control state, the peak was greater in control subjects than in diabetic patients in the distal gastric longitudinal muscle strips. The peak was decreased by the addition of atropine, and sequential addition of MRS2500, L-NNA, and TTX had no further effect on the peak. These observations indicate that the excitatory neural pathway, largely mediated by the cholinergic pathway, is also impaired in the diabetic distal stomach. Indeed, decreased expression of nNOS and SP accompanied by reduced ICC density was observed in the distal stomach of diabetics patients^6^. However, further studies are necessary to confirm our results.

The present study had some limitations. The clinical data of diabetic patients were limited, and diabetic gastropathy was not confirmed in them. In a population-based cohort study, the cumulative proportions developing gastroparesis over a 10-year period were 5.2% in type 1 DM, 1.0% in type 2 DM, and 0.2% in controls^26^. However, the prevalence of delayed gastric emptying in patients with diabetes has been reported to be between 28% and 65%^54,55^. In addition, a half of patients of diabetes have upper gastrointestinal symptoms^2,25^. Considering the long mean duration of disease (10.9 year) in our diabetic subjects, the observed differences in response to EFS of gastric muscles from diabetic and non-diabetic patients may contribute to the pathogenic mechanisms underlying diabetic gastropathy. Secondly, a majority of the diabetic patients had type 2 DM, and the sample size is rather small to be analyzed in terms of the differences in the stomach region and muscle layer. Nevertheless, to our knowledge, this is the first study comparing the response to EFS in normal and diabetic human stomach muscle. Therefore, our results may be of great importance for understanding the pathophysiology of diabetic gastropathy. In summary, diabetic patients have an impaired inhibitory nitrergic neural pathway in both the proximal and distal stomach and impaired excitatory cholinergic pathway in the distal stomach; these alterations may be the pathogenic mechanism of diabetic gastropathy.

## Materials and Methods

### Subjects and tissues

Gastric specimens were obtained from 34 diabetic patients (10 women, 24 men; mean age, 67.6 ± 6.3 [SD] years) and 45 control subjects (15 women, 30 men; mean age, 60.4 ± 11.9 years) who underwent gastrectomy for early gastric cancer at Samsung Medical Center, Seoul, Korea. The majority of diabetic patients (97%) had type 2 DM. The mean duration of DM and presence of glycated hemoglobin was 10.9 ± 7.1 years and 6.9% ± 0.9%, respectively. Control subjects did not have a history of DM. None of the control subjects had fasting serum glucose levels over 126 mg dL^−1^ or was using insulin or oral anti-hyperglycemic agents. No diffuse infiltrative type of gastric cancer was included in this study. None of the diabetic patients and control subjects had pre-operative radiotherapy, irritable bowel syndrome, or neurological disorders. They had no GI disease other than gastric cancer.

Shortly after gastrectomy, gastric muscle strips were excised from areas free of macroscopic evidence of cancer infiltration. The areas where the gastric tissues were removed from were mapped by the performing surgeon into the gastric fundus, corpus, or antrum. The surgeon was instructed regarding mapping of the exact gastric areas prior to the procedure^5^. The fundus was designated as the proximal stomach and the corpus and antrum as the distal stomach. Fourteen (6 circular and 8 longitudinal) gastric muscle strips from the proximal stomach and 46 (20 circular and 26 longitudinal) from the distal stomach were obtained in diabetic patients and 25 (11 circular and 14 longitudinal) from the proximal stomach and 41 (16 circular and 25 longitudinal) from the distal stomach were obtained in control subjects.

The excised tissues were collected in cold Krebs-Ringer bicarbonate (KRB) solution and transported to laboratory within 10 min after collection. KRB solution had the following composition (in mM): 120.4 NaCl, 5.9 KCl, 1.2 MgCl_2_, 15.5 NaHCO_3_, 1.2 KH_2_PO_4_, 11.5 dextrose, and 2.5 CaCl_2_. Tissues were pinned to the base of a Sylgard silicone elastomer (Dow Corning, Midland, MI) dish. The mucosa was removed by sharp dissection. After the mucosa was removed, thin strips of tissues were cut by use of parallel scalpel blades mounted on a scalpel handle. The final strips cut parallel to the muscle fibers measured 2 mm × 10 mm. The muscle strips were isolated and attached to a fixed mount and to a Fort 10 isometric strain gauge (UC3-GOULD Instruments, Paris, France; FT03-GFASS, Warwick, RI). The muscle strips were immersed in organ baths maintained at 37 ± 0.5 °C with oxygenated KRB solution. The pH of the KRB solution was 7.3–7.4 when bubbled with 97% O_2_-3% CO_2_ at 37 ± 0.5 °C. This preparation process was performed within 30 min after tissue collection.

The study protocol was conducted in accordance with the Declaration of Helsinki and approved by the Institutional Review Board at Samsung Medical Center, Seoul, Korea. All subjects provided written informed consent before inclusion in the study.

### Organ chamber experiments

Experiments were performed *in vitro* using strips of the human gastric muscle, and their mechanical activity was recorded as changes in isometric force. These experiments were conducted using standard organ bath techniques, as previously described in another study by our group^5^. The sample strips were equilibrated in the organ baths, which were continuously perfused with oxygenated KRB solution, for 60 mins. Based on our preliminary study (data not shown), all experiments were performed with an optimal degree of passive tension, which was 0.7 g. Changes in tension due to relaxation or contraction of the muscles were recorded through an analog-to-digital board to a computer (Chart5-ADInstruments, Bella Vista, NSW, Australia). Experiments were performed after basal tone was stabilized. Basal tone did not change through the entire experiment with repeated stimulations. Actual peak amplitudes were measured with adjusted baseline using Clampfit software (Molecular Devices, pClamp version 10, Sunnyvale, CA). The lowest value of stabilized spontaneous phasic contractions was defined as baseline (zero point). EFS-induced responses were measured with reference to the baseline. EFS (0.3 ms in trains of 10 Hz for 20 s, 150 V) was applied via two platinum ring electrodes attached to each strip. The electrodes were connected to a GRASS S88 (GRASS Instruments, Quincy, MA) stimulator.

### Protocols and measurements

**Figure 7:**
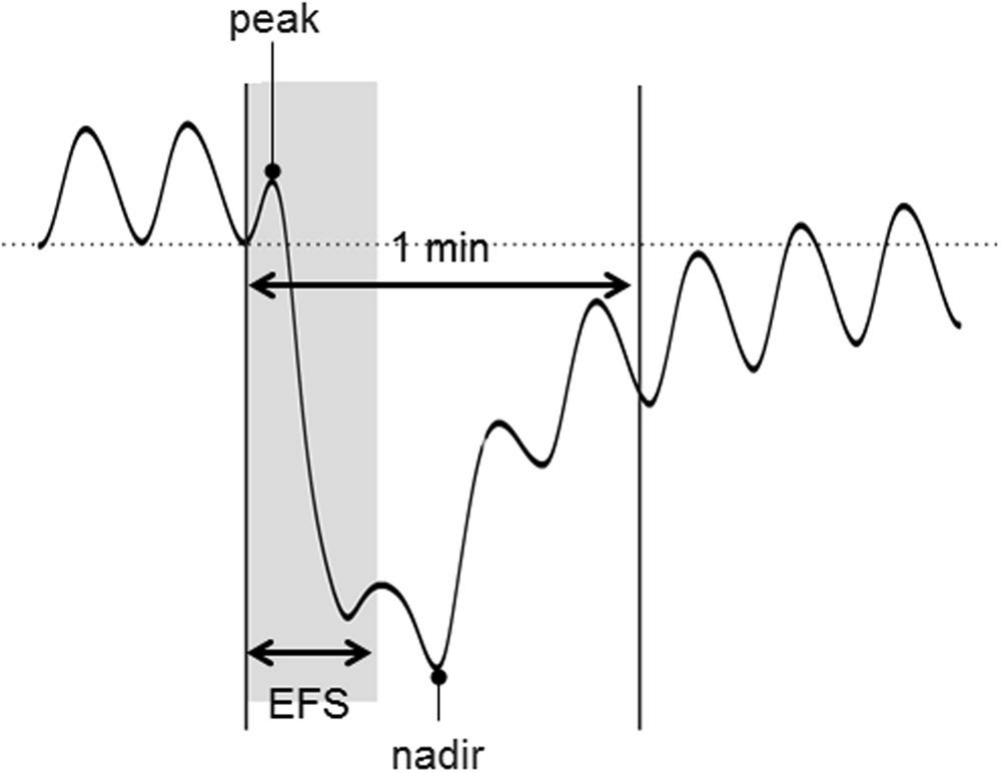
Peak and nadir of the response induced by electrical field stimulation (EFS) of human gastric muscle. Peak is the highest value, while nadir stands for the lowest value during the first 1 minute after the initiation of EFS.

**Figure 8:**
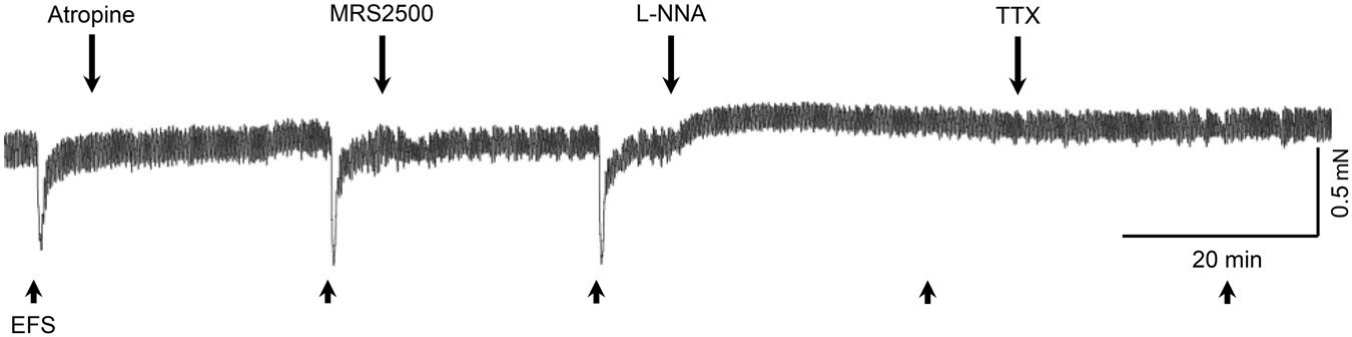
Typical tracing of electrical field stimulation (EFS)-induced response in gastric circular muscle strips with serial administration of atropine, MRS2500, N-nitro-L-arginine (L-NNA), and tetrodotoxin (TTX). Note the reduction of the relaxation by L-NNA.

We examined EFS-induced responses for the peak (the highest value) and nadir (the lowest value). The peak and nadir of both circular and longitudinal muscle strips were measured during the first 1 minute after the initiation of EFS (Fig. 7) in control state and after administration of atropine, MRS2500, and N-nitro-L-arginine, which were added in a sequential order to the organ bath (Fig. 8). Then, tetrodotoxin was used to confirm that the responses to EFS were mediated via neural stimulation. The muscle strips were sequentially perfused with each drug for 20 min after EFS and were allowed a recovery time of 5 min.

### Drugs and solutions

Atropine (Sigma Aldrich), MRS2500 (TOCRIS bioscience), L-NNA (Sigma Aldrich), and TTX (Alomone Labs) were used. Atropine, MRS2500, and TTX were dissolved in distilled water, while L-NNA was dissolved in 1 M hydrochloric acid. The stock concentration of L-NNA was 100 mM.

### Statistical analysis

Statistical analyses were conducted using SPSS version 21 (SPSS IBM, NY, USA). Experimental data are shown as mean ± SE. Mann-Whitney U-test was used to compare responses to EFS between control subjects and diabetic patients. Wilcoxon signed ranks test was used to evaluate changes in peak and nadir of EFS-induced contractile responses after sequential exposure to drugs. A two-sided *P* value < 0.05 was taken as statistically significant.
